# Complete chloroplast genomes of five classical Wuyi tea varieties (*Camellia sinensis*, Synonym: *Thea bohea* L.), the most famous Oolong tea in China

**DOI:** 10.1080/23802359.2022.2062263

**Published:** 2022-04-11

**Authors:** Li Fan, Li Li, Yunfei Hu, Yibiao Huang, Yongcong Hong, Bo Zhang

**Affiliations:** College of Tea and Food Science, Wuyi University, Wuyishan, People’s Republic of China

**Keywords:** Wuyi tea, chloroplast genome, phylogenetic analysis, super-barcode

## Abstract

Wuyi tea (*Camellia. sinensis*, Synonym: *Thea Bohea* L.) is recognized as the most prestigious oolong tea in China. For germplasm identification and protection, the complete chloroplast genomes of five classical Wuyi tea varieties were determined by next-generation sequencing. These chloroplast genomes showed highly conserved structures and are 157,024–157,126 bp in length, consisting of a pair of reverse repeats (IR) regions of 25,944–26,095 bp, one large single-copy (LSC) region of 86,594–86,859 bp, and one small single copy (SSC) region of 18,276–18,291 bp. A total of 137 genes were observed and overall GC contents were all about 37.3%. Phylogenetic analysis revealed Wuyi tea varieties did not cluster together, suggesting that these Wuyi tea varieties might have diverged early in their evolutionary history and the complete chloroplast genome could be used as a super-barcode to identify these varieties. This study will be valuable for future studies of evolution and intraspecific identification in Wuyi tea.

Wuyi tea belongs to *Camellia. sinensis* species. It usually named after the English transliteration (Bohea) of Wuyi, and was named *Bohea* or Var *Bohea* (*Thea bohea* Linnaeus. 1762, GBIF Secretariat 2019). Wuyi tea was cultivated in rocky soil of Wuyi Mountain, where is a UNESCO World Heritage site in northern China’s Fujian province and considered as the birthplace of Oolong tea. Recognized as the most prestigious oolong tea in China, Wuyi tea is renowned for its rich flavor and long-lasting fragrance, so-called ‘rock charm and floral fragrance’ (Lou et al. 2017; Chen et al. 2018). Wuyi tea has a long history of more than 1500 years, and there were thousands of varieties in history (Xiao 2017). Nowadays, due to the lack of effective protection, a large number of historical Wuyi tea varieties have been lost or difficult to find, causing irreversible losses. Therefore, it is urgent to protect and improve the germplasm resources of Wuyi tea.

The chloroplast (cp) genome can provide valuable information for phylogenetic studies and species identification (Bi et al. 2018). So far, only one cp genome of Wuyi tea has been reported (Li et al. 2021), which is not enough to provide effective molecular information to distinguish more rare varieties of Wuyi tea. Here, we present a complete assembly of cp genomes of five representative Wuyi tea cultivars, including *C. sinensis* cv. *Baijiguan* (BJG), *C. sinensis* cv. *Rougui* (RG), *C. sinensis* cv. *Shuijingui* (SJG), *C. sinensis* cv. *Tieluohan* (TLH) and *C. sinensis* cv. *Bantianyao* (BTY), which were known as their long history and good quality.

In this study, the young leaves of five cultivars were collected for genomic DNA isolation using the CTAB method. All five cultivars were originally from Wuyi Mountain, Fujian Province (27°43′42.46ʺN, 118°0′14.40ʺE) and then asexually propagated and planted in the Tea Germplasm Resources Museum of Wuyi University (The voucher number: BJG, No. 20200318; RG, No. 20200320; SJG, No. 20200321; TLH, No. 20200322 and BTY, No. 20200323). The genomic DNA were preserved at −80 °C at the Key Laboratory of Tea germplasm Genetic Resources of Wuyi University.

The complete cp genome was sequenced using a combination of PacBio RS and Illumina sequencing platforms by Biozeron Co., Ltd. (Shanghai, China). For PacBio sequencing, the average coverage reached 263×, 667×, 2370×, 1939×, 1665× and 2060× sequencing depth for five cp genomes assembly (BJG, RG, SJG, TLH and BTY), respectively. For Illumina sequencing, the average coverage reached 39313×, 30796×, 32018×, 36436× and 34766× sequencing depth, respectively. Briefly, PacBio and Illumina reads were mapped against the published cp genomes of *C. sinensis* var. *sinensis* (Accession number: KJ806281; Huang et al. 2014) to filter out the cp reads and be de-novo assembled using CLC Genomics Workbench 11.0.1. Cp genes were annotated by cpGAVAS (Liu et al. 2012) and sequenced coordinates of annotated cp genes available on NCBI using BLAST search (Acland et al. 2014). The final annotated cp genome sequences were deposited to NCBI GenBank (Accession number: BJG, MT773373; RG, MT773375; SJG, MT773376; TLH, MT773377 and BTY, MW046255).

The five cp genome lengths ranged from 157,024 bp (BTY) to 157,126 bp (SJG). The cp genomes consisted of circular double-stranded DNA and showed a typical quadripartite structure, including an LSC region of 86,594–86,859 bp, an SSC region of 18,276–18,291 bp, and a pair of the IR regions of 25,944–26,095 bp. A total of 137 coding genes were identically annotated in the same order, consisting of 92 protein-coding genes, 37 tRNA genes, and 8 rRNA genes. Except for two introns in the ycf3 and clpP genes, all other genes contained only one intron. Overall GC contents were all about 37.3%. To explore the phylogenetic positions and evolutionary relationships of Wuyi tea varieties, 46 *Camellia* species and an outgroup *(Apterosperma oblata) w*ere selected for the construction of Bayesian tree (Figure 1). The best-fitting models were determined by the GTR as implemented in Modeltest 3.7. Bayesian analyses were performed with four independent chains running for two million generations, sampling a tree every 100 generations, the first 2500 trees were removed as burn-in and the remaining trees were used calculate Bayesian posterior probabilities. The result showed Wuyi tea cultivars did not cluster together, but intermingled with other *C. sinensis* var. *sinensis* varieties, reflecting the rich genetic diversity of Wuyi tea varieties. All these Wuyi tea cultivars could be effectively distinguished with high support, suggesting that Wuyi tea cultivars might have diverged early in their evolutionary history and the complete cp genome can be used as a super-barcode to identify them.

**Figure 1.**
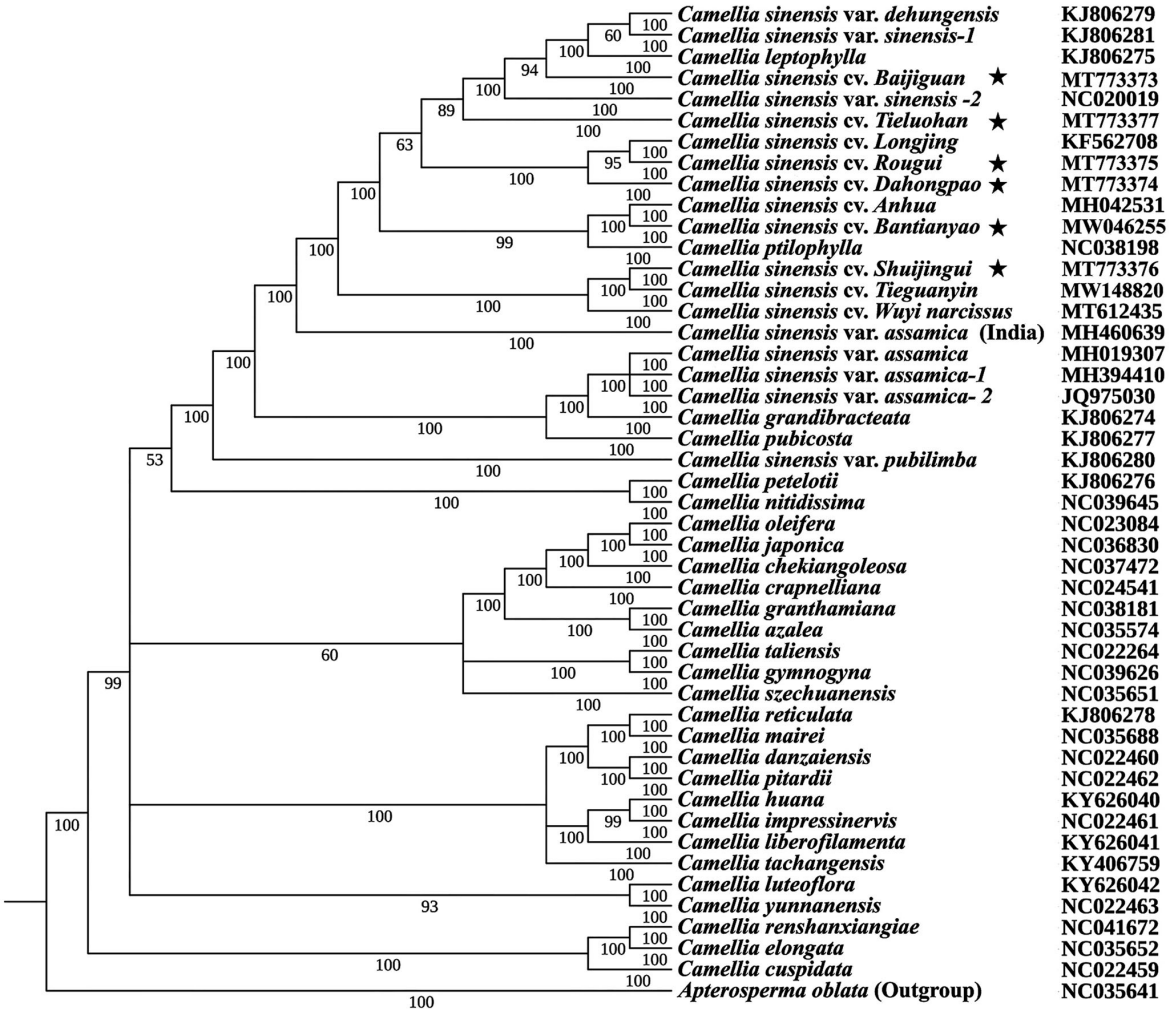
Phylogeny of *Camellia sinensis* based on the complete chloroplast genome. Numbers associated with nodes indicated Bayesian inference (BI) posterior probabilities values. The position of Wuyi tea varieties were marked with black asterisk and GenBank number was listed behind each variety name.

## Data Availability

The genome sequence data that support the findings of this study are openly available in GenBank of NCBI at (https://www.ncbi.nlm.nih.gov/) under NCBI accession number: MT773373 (BJG), MT773375 (RG), MT773376 (SJG), MT773377 (TLH) and MW046255 (BTY). The associated BioProject numbers are PRJNA644760 (BJG, RG, SJG, TLH) and PRJNA666051 (BTY). The associated SRA numbers are SRS7200201 (BJG), SRS7200203 (RG), SRS7200204 (SJG), SRS7200205 (TLH), and SRS9680346 (BTY). The associated Bio-Sample numbers are SAMN15804988 (BJG), SAMN15804990 (RG), SAMN15804991 (SJG), SAMN15804992 (TLH), and SAMN20564595 (BTY).
